# Anti-Neutrophil Cytoplasmic Antibody (ANCA)-Associated Fasciitis Mimicking Pseudogout in an Older Patient: A Diagnostic Challenge and Treatment Approach

**DOI:** 10.7759/cureus.71585

**Published:** 2024-10-16

**Authors:** Natsumi Yamamoto, Ryuichi Ohta, Akira Yamasaki, Chiaki Sano

**Affiliations:** 1 Department of Community Care, Unnan City Hospital, Unnan, JPN; 2 Division of Respiratory Medicine and Rheumatology, Department of Multidisciplinary Internal Medicine, Faculty of Medicine, Tottori University, Yonago, JPN; 3 Department of Community Medicine Management, Shimane University Faculty of Medicine, Izumo, JPN

**Keywords:** anca-associated vasculitis, autoimmune diseases, elderly, family medicine, fasciitis, general medicine, immunosuppressive therapy, prednisolone, rural

## Abstract

ANCA-associated fasciitis is a rare manifestation of ANCA-associated vasculitis (AAV) that can mimic other conditions, such as pseudogout, especially in elderly patients. We present the case of a 93-year-old woman who initially developed polyarthralgia, muscle pain, and difficulty walking, symptoms suggestive of pseudogout. However, after further investigation, including elevated myeloperoxidase (MPO)-ANCA levels and MRI findings, she was diagnosed with ANCA-associated fasciitis. Treatment with high-dose prednisolone and rituximab led to significant clinical improvement. This case highlights the diagnostic challenges of ANCA-associated fasciitis and emphasizes the importance of a comprehensive approach for early diagnosis and management to prevent functional decline.

## Introduction

Anti-neutrophil cytoplasmic antibody (ANCA)-associated vasculitis (AAV) is a group of autoimmune diseases characterized by inflammation of blood vessels, which can damage various organs [1]. Among its manifestations, ANCA-associated fasciitis is a rare and underrecognized condition, particularly in elderly patients [2]. While AAV typically presents with renal and pulmonary involvement, the occurrence of fasciitis, especially as an isolated symptom, poses diagnostic challenges [3,4].

This case report describes an older woman with ANCA-associated fasciitis, an uncommon and challenging manifestation of AAV characterized by atypical symptoms that delay diagnosis. The absence of typical organ involvement, such as renal or pulmonary manifestations, complicated the clinical picture. This case emphasizes the need for clinicians to consider AAV in the differential diagnosis of fasciitis, even when classical features are absent. It aims to raise awareness of this rare presentation and stresses the importance of a comprehensive diagnostic approach and early recognition to avoid misdiagnosis and provide timely treatment.

## Case presentation

A 93-year-old woman visited a regional referral hospital with a chief complaint of polyarthralgia. Initially, she had been living independently, but ten days before coming to the hospital, she started experiencing acute onset pain in both buttocks without any trauma. The pain in both buttocks worsened, and it gradually became difficult for her to move. As a result, she was transported by emergency services for treatment. There was no fever, night sweats, chills, weight loss, chest pain, or abdominal pain. Her medical history included brainstem hemorrhage, reflux esophagitis, hypertension, overactive bladder, and constipation. Her medications included olmesartan 20 mg daily, lansoprazole 15 mg daily, mirabegron 50 mg daily, magnesium oxide 990 mg daily, and elobixibat 10 mg daily.

Upon admission, she was conscious. Her vital signs were as follows: temperature 36.6°C, blood pressure 139/66 mmHg, pulse 88 beats per minute, peripheral capillary oxygen saturation (SpO2) 90% on 1 L/min of oxygen, and respiratory rate 19 breaths per minute. Physical examination revealed late inspiratory crackles at the lung bases on both sides and a systolic murmur radiating to the neck. Joint findings included heat and tenderness in the shoulders, elbows, hips, and knees. There was grasping pain in both necks, thighs, and forearms. Erythema was observed around the umbilicus. Blood tests showed increased C-reactive protein (CRP) (3.21 mg/dL) and lactate dehydrogenase (LDH) (525 U/L) (Table 1).

**Table 1:** Initial laboratory data for the patient. CRP: C-reactive protein; eGFR: Estimated glomerular filtration rate; Ig: Immunoglobulin; PT: Prothrombin time; INR: International normalized ratio; APTT: Activated partial thromboplastin time.

Parameter	Level	Reference
WBCs	7.0	3.5-9.1 × 10^3^/μL
Neutrophils	66.8	44.0-72.0%
Lymphocytes	15.7	18.0-59.0%
Hemoglobin	12.3	11.3-15.2 g/dL
Hematocrit	36.8	33.4-44.9%
Mean corpuscular volume	88.4	79.0-100.0 fl
Platelets	23.0	13.0-36.9 × 10^4^/μL
Erythrocyte sedimentation rate	18	2-10 mm/hour
Total protein	6.5	6.5-8.3 g/dL
Albumin	3.2	3.8-5.3 g/dL
Total bilirubin	0.9	0-1.2 mg/dL
Aspartate aminotransferase	34	8-38 IU/L
Alanine aminotransferase	23	4-43 IU/L
Lactate dehydrogenase	525	121-245 U/L
Blood urea nitrogen	21.7	8-20 mg/dL
Creatinine	0.59	0.40–1.10 mg/dL
eGFR	69.3	ml/min/L
Creatinine kinase	168	43-165 U/L
Serum Na	140	135–150 mEq/L
Serum K	4.4	3.5–5.3 mEq/L
Serum Cl	107	98–110 mEq/L
Serum Ca	9.2	8.8-10.2 mg/dL
CRP	3.21	<0.30 mg/dL
Immunoglobulin G	1166	870–1700 mg/dL
Immunoglobulin M	64	35–220 mg/dL
Immunoglobulin A	275	110–410 mg/dL
PT (%)	106	70-130 %
PT-INR	0.97	
APTT (seconds)	30.2	25-40 seconds
D-dimer (μg/mL)	5.4	<1.00 μg/mL
Urine test	-	-
Leukocyte	Negative	Negative
Protein	Negative	Negative
Blood	Negative	Negative

**Figure 1:**
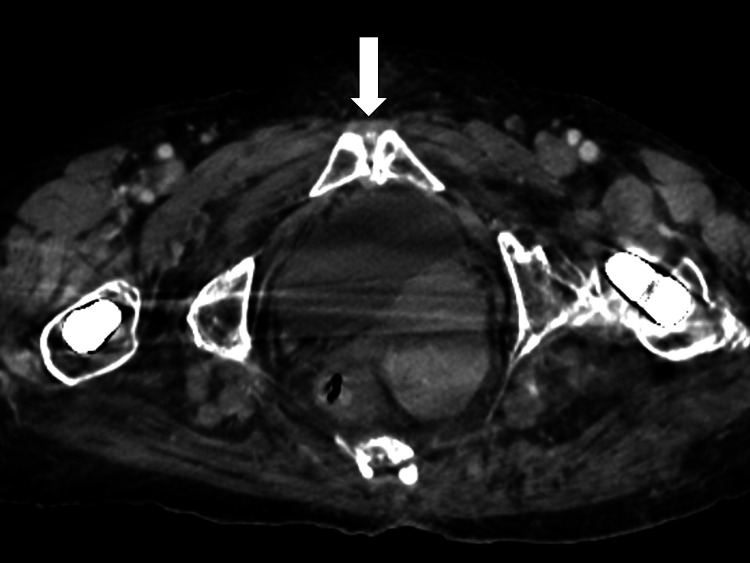
A chest-to-pelvic computed tomography image revealing calcification around the shoulder joint and the pubic symphysis (indicated by white arrows).

As a differential diagnosis for systemic pain, we considered the possibility of a deep abscess. A chest-to-pelvic CT scan was performed, which revealed calcification around the shoulder joint, calcification around the pubic symphysis, and a multilocular cyst in the right ovary (Figure 1).

There was no evident abscess formation.

Based on the above, considering systemic pain due to cellulitis around the umbilicus, we started Ampicillin Sodium Sulbactam Sodium at 6g daily, and for polyarticular pain and myalgia, we started Loxoprofen at 180mg daily as treatment for multiple pseudogout. On the third day after admission, fever persisted, as did polyarticular pain and myalgia. There was no response to Loxoprofen, so we switched from Loxoprofen to prednisolone (PSL) at 20 mg daily.

**Figure 2:**
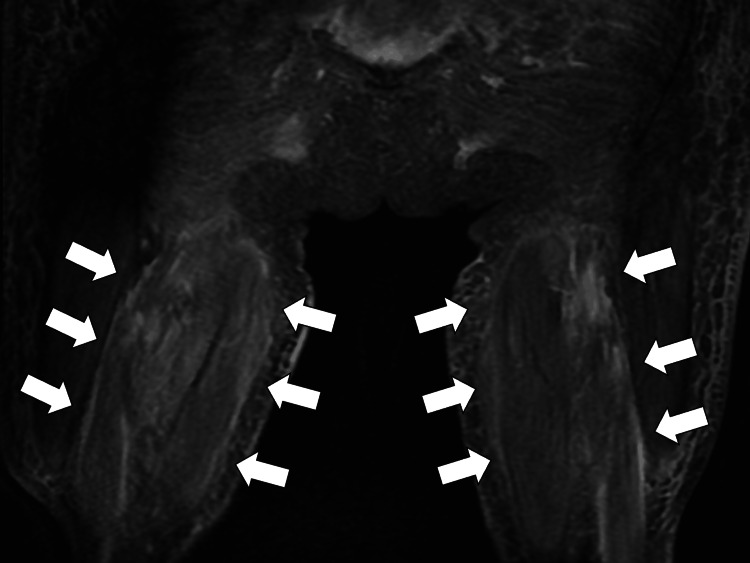
Lower limb muscle MRI (short tau inversion recovery) showing high signal intensity in bilateral quadriceps and biceps femoris (indicated by white arrows).

Since the symptoms persisted even after treatment for the infection and there were no obvious sepsis-like symptoms, we considered the possibility of autoimmune disease, including myositis and vasculitis, as a differential diagnosis. Autoantibodies were submitted for blood tests, and wrist X-ray and lower leg MRI were added for further investigation. Antinuclear antibody (ANA), rheumatoid factor (RF), and anti-citrullinated peptide antibody (ACPA) were negative, and MPO-ANCA were elevated at 94.4 U/mL [reference, <3.5 U/mL]. The wrist X-ray showed no erosion or narrowing of the joint space. Lower limb muscle MRI showed atrophy toward the inner dorsal side of the thigh. Short τ inversion recovery (STIR) images showed high signal intensity, fat suppression images showed high signal intensity, and edematous and inflammatory changes were observed in bilateral quadriceps and biceps femoris (Figure 2).

**Figure 3:**
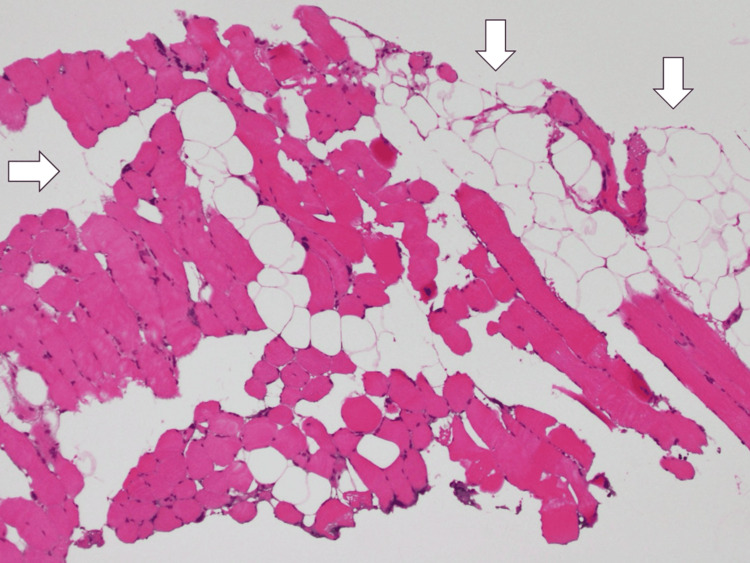
The biopsy of the right biceps femoris showing the muscle’s atrophy with fatty infiltration (white arrows).

A thigh muscle biopsy was also performed, and the biopsy result only showed the muscles' atrophy with fatty infiltration (Figure 3).

Based on the above results, the patient was diagnosed with muscle-localized ANCA-associated vasculitis based on the European League Against Rheumatism (EULAR) criteria of AAV, and PSL at 55 mg was started on the 6th day of hospitalization [5]. Furthermore, as induction therapy, weekly drip administration of Rituximab 500 mg was started on the 11th day of hospitalization and continued for four weeks.

After starting treatment, polyarticular pain and myalgia improved, and inflammatory conditions also improved. ANCA also turned negative, and steroids were gradually tapered, and Rituximab was administered. She was moved to a rehabilitation ward, and her rehabilitation was intensified in preparation for discharge.

## Discussion

This case report highlights older patients presenting with polyarthritis, muscle pain, and difficulty walking who were eventually diagnosed with ANCA-associated vasculitis (AAV) fasciitis, mimicking pseudogout. It suggests that older patients with polymyalgia and polyarthritis require precise monitoring of symptoms and clinical courses to adjust treatment intensity and reconsider diagnoses. In community hospitals, where older patients exhibit multiple vague symptoms, general physicians should be vigilant about subtle changes in symptoms to accurately diagnose and prevent frailty from critical diseases such as AAV.

As part of the diagnostic process for ANCA-related fasciitis, the diagnostic accuracy of fascial biopsy is low, necessitating a comprehensive approach based on exclusionary diagnoses [6]. Pathological diagnosis is crucial for diagnosing ANCA-associated vasculitis, but subtle changes caused by ANCA may be difficult to detect with a biopsy [7]. If the lesion site in the fascia biopsy is hard to visually identify, as in this case, the pathological results may not be definitive [8]. Furthermore, the symptoms of ANCA-related myositis may progress rapidly [2]. Promptly ruling out a fatal disease and starting multidisciplinary treatment early can prevent a decline in ADL and lead to early hospital discharge [9,10].

Treatment strategies for ANCA-associated fasciitis are critical, and PSL and rituximab should be used as soon as clinical diseases are excluded. Diagnosing, treating, or excluding sepsis is crucial in this context. Symptoms of sepsis are nonspecific in older patients [11,12]. When general physicians observe the progression of systemic symptoms, it is vital to administer antibiotics immediately to the potentially infected organs. Additionally, this case was complicated by polyarthritis, and the possibility of pseudogout could not be ruled out [13]. We started with a low dose of PSL. Generally, pseudogout goes into remission with 10-15 mg of PSL. After treating sepsis, if the patient does not achieve remission with a low dose of PSL, it is necessary to proactively consider the presence of vasculitis [13].

In diagnosing ANCA-associated fasciitis, the accuracy of fascial biopsy is low, and a comprehensive approach based on differential diagnosis is necessary. Although pathological diagnosis is important in diagnosing ANCA-associated vasculitis, the subtle changes caused by ANCA may be difficult to detect through biopsy [14]. As observed in the present case, pathological findings may be negative when it is challenging to visually identify the lesion during a fascial biopsy [15]. Moreover, the symptoms of ANCA-associated myositis can progress quickly [9]. After promptly excluding life-threatening diseases, initiating multidisciplinary treatment as early as possible can help prevent a decline in ADL and facilitate earlier discharge.

Regarding the treatment strategy for ANCA-associated fasciitis in older patients, once critical conditions are excluded, it is crucial to smoothly initiate therapy using PSL and rituximab. In this case, diagnosing, treating, or excluding sepsis was significant. Symptoms of sepsis in elderly patients tend to be non-specific. As observed in this case, when systemic symptoms progress, it is essential to first focus on any potentially infected organs and administer appropriate antibiotics for treatment [16]. Furthermore, the patient also had polyarthritis, and the possibility of pseudogout could not be excluded. Therefore, low-dose PSL was initiated. Generally, pseudogout inflammation is considered to remit with PSL at doses of 10-15 mg/day. In cases like the present one, if remission is not achieved with low-dose PSL after addressing sepsis, it is necessary to actively consider the presence of vasculitis and take appropriate action.

In rural community hospitals, for significantly older patients presenting with fever and polyarthritis, it is essential to first conduct a thorough evaluation for infections and initiate treatment accordingly. If there is no response to antibiotics or if sepsis-like symptoms are absent, autoimmune diseases should be suspected, and tests for ANCA, RF, anti-CCP antibodies, and antinuclear antibodies should be performed [17]. Additional imaging should be conducted if there are localized physical findings [18]. While biopsy may be attempted when feasible, it is not always necessary; as demonstrated in this case, it may be possible to confirm localized inflammatory findings through MRI [19]. It is important not to rely too heavily on pathological diagnosis but to initiate early treatment to prevent a decline in ADL caused by symptoms.

## Conclusions

This case highlights the diagnostic complexity of ANCA-associated fasciitis, particularly in older patients with atypical presentations. The rarity of this condition and its mimicry of other diseases, such as pseudogout, underscore the need for general physicians to maintain a high index of suspicion. Early recognition and a comprehensive, multidisciplinary approach to diagnosis and treatment are critical for preventing functional decline and ensuring better patient outcomes. Prompt initiation of immunosuppressive therapy, including prednisolone and rituximab, after ruling out infections can lead to symptom improvement and facilitate early recovery.
